# The microbiome of glaciers and ice sheets

**DOI:** 10.1038/s41522-017-0019-0

**Published:** 2017-04-19

**Authors:** Alexandre M. Anesio, Stefanie Lutz, Nathan A. M. Chrismas, Liane G. Benning

**Affiliations:** 1 Bristol Glaciology Centre, School of Geographical Sciences, University of Bristol, Bristol, BS8 1SS UK; 2 GFZ German Research Centre for Geosciences, Telegrafenberg, 14473 Potsdam, Germany; 3 Department of Earth Sciences, Free University of Berlin, 12249 Berlin, Germany

## Abstract

Glaciers and ice sheets, like other biomes, occupy a significant area of the planet and harbour biological communities with distinct interactions and feedbacks with their physical and chemical environment. In the case of the glacial biome, the biological processes are dominated almost exclusively by microbial communities. Habitats on glaciers and ice sheets with enough liquid water to sustain microbial activity include snow, surface ice, cryoconite holes, englacial systems and the interface between ice and overridden rock/soil. There is a remarkable similarity between the different specific glacial habitats across glaciers and ice sheets worldwide, particularly regarding their main primary producers and ecosystem engineers. At the surface, cyanobacteria dominate the carbon production in aquatic/sediment systems such as cryoconite holes, while eukaryotic Zygnematales and Chlamydomonadales dominate ice surfaces and snow dynamics, respectively. Microbially driven chemolithotrophic processes associated with sulphur and iron cycle and C transformations in subglacial ecosystems provide the basis for chemical transformations at the rock interface under the ice that underpin an important mechanism for the delivery of nutrients to downstream ecosystems. In this review, we focus on the main ecosystem engineers of glaciers and ice sheets and how they interact with their chemical and physical environment. We then discuss the implications of this microbial activity on the icy microbiome to the biogeochemistry of downstream ecosystems.

## Introduction

Glaciers and ice sheets have recently been recognised as one of the biomes on Earth.^1^ Biologically, these icy ecosystems are exclusively microbially driven, which is one of their unique features compared to other terrestrial biomes (e.g., tundra, tropical forests). One of the major limiting factors for the development of microbial processes is the presence of liquid water. The overwhelming presence of snow and ice does not preclude glaciers and ice sheets of containing large amounts of surface and interstitial water for long periods during the melt season. The annual runoff from Greenland alone is estimated to be around 10^15^ litres,^2^ while the amount of water stored as ice and snow globally is around 10^19^ litres. Bacterial cell abundances in the wet areas of glaciers vary significantly between 10^7^ cells L^−1^ in clean snow and ice^3^ to 10^11^ cells L^−1^ in surface ice associated with impurities.^4, 5^ We know little about the rate or types of microbes delivered onto remote snow and ice fields through Aeolian processes^6–8^ and about microbial abundances in subglacial habitats as these are far more difficult to sample. For subglacial systems, recent measurements of bacterial numbers associated with sediments from glaciers in the Arctic and Antarctica and subglacial runoff range between 0.87 and 7.9 × 10^6^ cells g^−1^ (ref. 9). It is estimated that glaciers and ice sheets around the globe can contain as many as 10^29^ cells.^10^ Thus, the amount of microorganisms stored and delivered from glaciers and ice sheets is far from trivial.

**Fig. 1:**
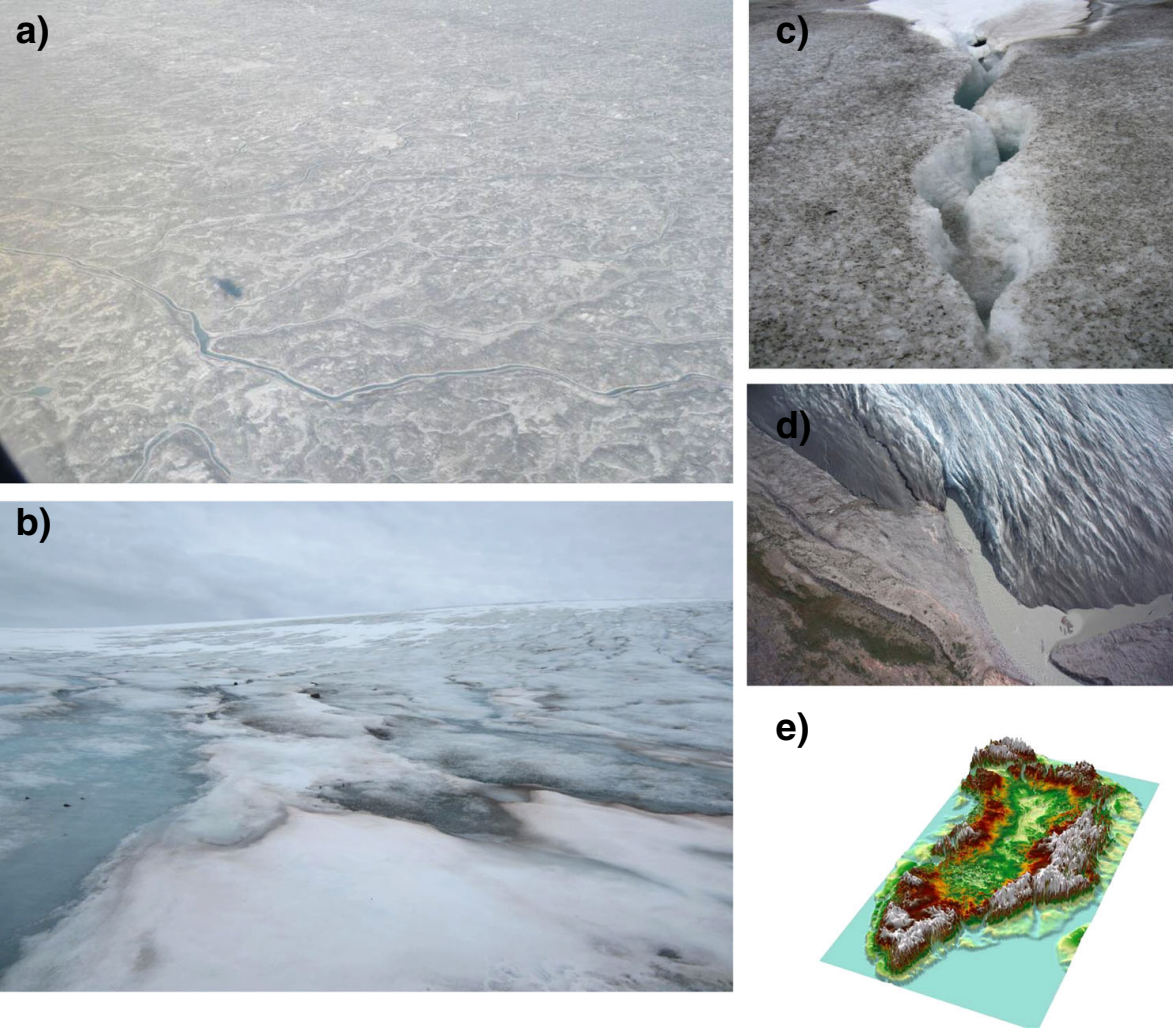
The different shades of colours and the glacial biome habitats. **a** Landscape scale view of the west side of the Greenland ice sheet (GrIS) showing the extent of impurities and colonisation of algae at the ice surface; **b** Photo of a section of the Mittivakkat glacier in the southeast coast of Greenland showing a variety of different surface habitats, from clean snow to “dirty” ice; **c** a small crevasse on the GrIS. Features like this provide opportunities for transport of material from the ice surface to the englacial system; **d** runoff from Leveret glacier (west Greenland—Kangerlussuaq) containing a large signature of subglacial water (sediment rich); **e** 3D visualisation of the canyon under the GrIS (Photo by J. Bamber based on ref. 135). The amount of water stored under the ice is unknown

**Fig. 2:**
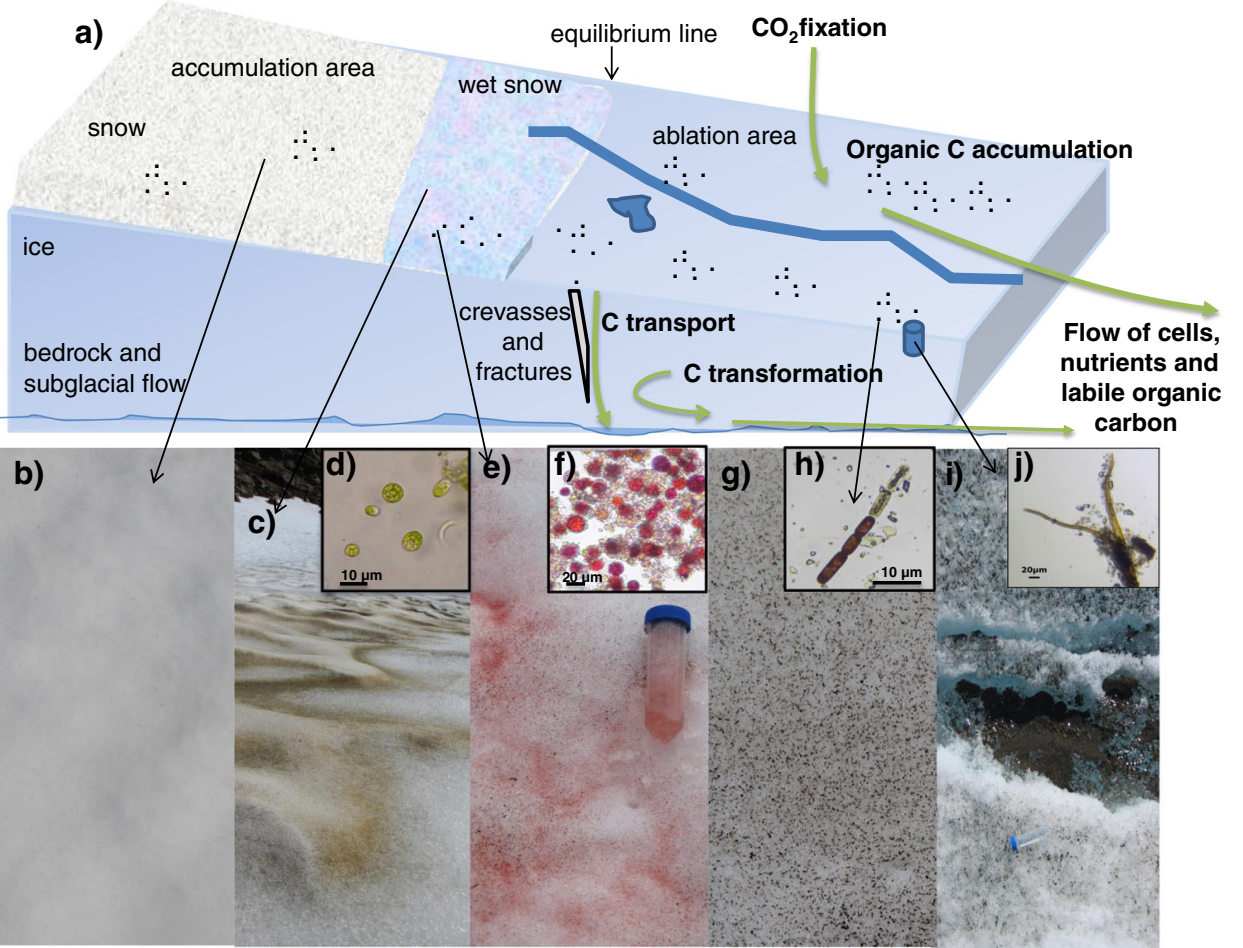
**a** Schematic figure of the different glacial habitats for biological colonisation and activity with a closer view of the surface ecosystem. The significance of microbial activity in englacial and subglacial systems is still largely unknown; **b** white snow at the beginning of the melt season; **c**, **d** green snow with its dominant main primary producer (Chlamydomonadales) collected from a Svalbard glacier; **e**, **f** red snow with its dominant main primary producer (Chlamydomonadales) collected from a Svalbard glacier, **g**, **h** “dirty” ice with one of its dominant main primary producer (a Zygnematales); **i**, **j** an example of a cryoconite hole in the GrIS and one of its main primary producers (*Phormidesmis priestleyi*)

In principle, any of the wet habitats in/on glaciers and ice sheets can have an active community of microorganisms. The amount and characteristics of these wet habitats can vary in both time and space. Wet snow can change its aspect during the summer melt season from white (Figs 1a, b and 2a) to different shades of green (Fig. 2c, d) and red (Fig. 2e, f) due to the presence of snow algae.^11^ Likewise, the ice surface can contain only very few impurities and be perfectly white or be characterised by the presence of large amounts of organic and inorganic impurities that also darken its surface^12^ (Figs 1a–c and 2g–h). Any such impurities, regardless if biological or inorganic in nature (which in turn can be both allochthonously delivered or autochthonously grown/recycled on site), provide nutrients for the growth of snow (mainly dominated by Chlamydomonadales) and ice algae (mainly dominated by Zygnematales) and heterotrophic bacteria. Depending on the amount of impurities, these dark features can melt into ice forming cryoconite holes (Fig. 2i–j). Such cryoconite holes can range from millimetres to tens of centimetres,^13^ and they are rich oases of microbial life and sites for active biogeochemical cycling on ice sheets and glaciers. The majority of microbial biomass in cryoconite holes is found within the cryoconite granules themselves^14^ and these tiny ecosystems are hosts to a complex consortium of algae, prokaryotic photoautotrophs and heterotrophs, and viruses. Important components of these communities in both polar and alpine ecosystems are the cyanobacteria.^15^

Due to the background geothermal heat and the pressure of the ice that keeps liquid water above the pressure melting point, water can also form beneath glaciers and ice sheets (Figs 1e and 2a). These ecosystems are dark and, depending on their hydraulic residence times, likely oxygen depleted.^16^ Samples collected from subglacial ice reveal prokaryote communities adapted to a range of redox conditions.^17^ In contrast, the englacial system (i.e., the habitat within the glacial ice—Figs 1d and 2a) is far less understood compared to any of the other habitats found at the surface and under the ice, yet prokaryotic organisms have also been found within ice, but very little is known about their function.^18^ This means that most knowledge about the glacial microbiome comes from a 2-D perspective (i.e., the surface and the interface between the bedrock and the ice).

Biomes on Earth are often described and characterised by the dominant vegetation that are uniquely adapted for the temperature and water availability conditions of that biome, providing the base for food webs in those large scale ecosystems. On glacial surfaces, snow and ice alga and cyanobacteria are the dominant primary producers and ecosystem engineers.^1^ They are responsible for the accumulation of organic matter at the surface of glaciers, which in turn leads to positive feedbacks between melting and microbial activity. Organic matter produced at the surface of the snow and ice by those ecosystem engineers can also be transported through the englacial system down to subglacial environments, fuelling microbial processes under the ice. In subglacial environments, several chemolithotrophic processes have been described providing an additional pathway by which microbial heterotrophic activity is sustained. In this review, we give particular emphasis on the primary producers of different glacial habitats.Several recent reviews have described the microbial ecology of the different habitats of glaciers and ice sheets (e.g., refs 1, 13, 19–22). Here, the main focus lies with the ecosystem engineers of glaciers and ice sheets (i.e., their primary producers) and how the organic matter produced and accumulated by them can modify their physical and chemical environment.

## Algae in snow

Snow algae are a group of unicellular freshwater algae (mainly *Chlorophyceae*) that thrive in glacial snow and permanent snow fields (Figs 1b and 2a–f). Snow algae taxa have been described in a plethora of polar and alpine settings (e.g., Svalbard,^23–27^ Greenland,^4^ Alaska,^28^ Iceland,^29^ the European Alps,^30^ the Himalayans,^31^ the Rocky Mountains,^32^ the Atlas Mountains^33^ and Antarctica^34^), suggesting a cosmopolitan occurrence for these organisms. The first descriptions of snow algal taxonomy, ecology and physiology were based on classical microscopy observations by Kol^35^ and Ettl^36^ and later by Hoham et al.^37^ and Komárek and Nedbalová.^38^ The red snow (sometimes also termed ‘‘watermelon snow’’) phenomenon has been known for a long time and has already been described by the Ancient Greek Aristotle.^39^ Yet, only in the 19th century the development of microscopy allowed scientists to link red snow to biological processes, namely snow algae blooms, and this work led to the naming of the taxon *Chlamydomonas nivalis* by Wille.^40^ Since then a large number of snow algal taxa have been described and wrongly classified as *Chlamydomonas nivalis*. Kol^35^was the first to state that this taxon has to be regarded as a collective name and that it is not the only species that causes the colouring. The same confusion applies for the taxon *Chloromonas nivalis* that cannot be regarded as one species either, as revealed by genetic analyses.^23^ Although important, this classification problem is still often neglected nowadays.

Snow algae can form extensive blooms in summer after the onset of melting. In response to extensive light exposure, they develop pigmentation as an adaptation mechanism. Depending on the concentration and composition of the produced pigments, the result is a macroscopically visible colouration of the snow. This can vary from green (chlorophylls) to different shades of yellow (xantophylls), orange and red (secondary carotenoids, i.e., astaxanthin).^11, 41^ Carotenoid-rich resting stages of *Chlamydomonas* and *Chloromonas* taxa are the typical causers of red snow mass blooms. In contrast, green snow can be both, a transient stage caused by the (still) chlorophyll-rich trophic stages of the latter red snow taxa^37, 42^ or an independent phenomenon caused by the genera *Microglena* ^27^ or *Raphidonema*.^35^ However, *Raphidonema sempervirens* and *R*. *nivale* (*Trebouxiophyceae*) are typical permafrost algae and not true snow algal taxa. Laboratory experiments^43^ also demonstrated that *Raphidonema sempervirens* produces primary (i.e., xantophylls), but not secondary (i.e., astaxanthin) carotenoids, that are characteristic of the red snow cysts. *Raphidonema sempervirens* use the xanthophyll cycle to dissipate excessive energy as heat through non-photochemical quenching.^44^ Furthermore, *Raphidonema* cells are likely introduced onto glacial surfaces by wind rather than through in situ propagation.^45^ Thus, it remains unclear, whether this taxon is a critical or a minor player in the ecology of glaciers. Finally, less often described (and probably less abundant) is yellow snow caused by *Hydrurus* related *Chrysophyceae*.^46^

The parameters controlling snow algal distribution are far from being well understood. Several studies^25, 27, 29, 35, 47^ concluded that ecosystem chemistry may only play a minor role in comparison to physical parameters, which includes field topography (e.g., slope) that impacts on melting. Using molecular identification, Lutz et al.^47^ found similar snow algal community composition across the Arctic that is independent of location-specific geochemical factors, with an uncultured Chlamydomonadaceae taxon showing the highest relative abundance. On the other hand, Brown et al.^48^ showed that on the haplotype level, snow algal populations are locally heterogeneous and each population was derived from one clone. Whereas at the genus and most often species level the above mentioned taxa can be found in the Arctic, Antarctic and the European Alps, future work needs to investigate their similarities or differences on the sub-species level as well as their distribution patterns on different scales (e.g., local, regional, global).

The main stages in snow algal life cycles have been described in Hoham et al.^37^ and Remias,^42^ who suggested that *Chlamydomonas nivalis* cells remain dormant as cysts for most of the year. At the onset of melting in spring flagellated cells emerge and migrate to the snow surface where they start to bloom. However, this migration process has not yet been fully observed or documented in field settings and needs further investigation. During the melt season, cells undergo a variety of intracellular rearrangements and changes in morphology (which make species identification by microscopy very challenging). These stages are likely linked to nutrient limitation, as under nitrogen-deficiency the metabolism is directed towards nitrogen-free metabolites such as lipids (e.g., fatty acids) and pigments (e.g., secondary carotenoids).^43^ Astaxanthin, the main secondary carotenoid, is often esterified with fatty acids and stored in cytoplasmatic lipid droplets around the nucleus and chloroplast, which in turn also results in their shading from high light irradiation.^49^ Thus, protection against excessive radiation could also involve the accumulation of primary and secondary carotenoids (to shield the chloroplast), which enhances lipid production (less cytoplasmatic water content), resulting in reinforcement of the cell wall and cyst formation as one of the suit of adaptation mechanisms for organic carbon production in snow. Snow algae are actively fixing carbon in these ecosystems as demonstrated by net ecosystem production measurements.^4^ The potential for labile organic carbon production in snow is reflected in bacterial abundance and production rates, that were found to be higher in red snow containing algae compared to white snow with low snow algal numbers.^50^ This suggests that snow algae are an important source of organic matter. Similarly, Brown et al.^51^ found a co-occurrence of snow algae and fungi but no studies have been conducted to investigate the nature of their interaction.

True snow algae are psychrophiles with a temperature optimum below 15 °C, maximum temperature around 20 °C and the preferred growth temperature around 0 °C. Many cryophilic adaptation strategies can only be found in truly psychrophilic snow algae,^37^ and these are of wide interest for biotechnological application.^52^ A large number of cultured snow algal strain is available from CCCryo (Culture Collection of Cryophilic Algae http://cccryo.fraunhofer.de/web/infos/welcome/), which serves as a valuable bioresource for laboratory experiments (e.g., ref. 43).

With the rise of next-generation sequencing techniques and their broad application in many other environments, it is striking how little such approaches have been used for snow algae. Only very few studies have recently used ‘‘omics’’ techniques, including amplicon DNA sequencing,^4, 11, 29, 48, 51^ metagenomics^27^ and metabolomics^27^ to address diversity and functional traits in snow microbiomes. A major restriction is likely the lack of suitable snow algae reference genomes. The closest related genome available is that of the well-studied mesophile *Chlamydomonas reinhardtii*,^53^ which, however, does not share the cryophilic properties.

Important in a global context is the fact that pigmented snow algal blooms (together with the ice algae blooms—described below) decrease the albedo of snow and ice surfaces, which in turn may speed up melting processes.^4, 28, 50, 54^ Because, the availability of liquid water is one of the key drivers for snow algal growth, as a consequence of global warming, the frequency of extreme melt events will increase,^55, 56^ and likely intensify the distribution and duration of snow algal blooms, and in turn melt rates.

## Algae on ice surfaces

Less striking to the observer’s eye compared to the “red snow” phenomenon, but probably even more important on ice surfaces, are ice algal blooms (Figs 1a–c and 2g–h). Together with anthropogenic black carbon and mineral debris inputs,^8^ they impart glacial surfaces a brownish-greyish colouration that is often mistaken for ‘‘dirt’’. Although these blooms had already been mentioned in the 1870s by Adolf Erik Nordenskiöldii and Sven Berggren during expeditions to Greenland,^57, 58^ ice algae have only recently been described in detail.^59–61^ Ice algal mass blooms are believed to show cosmopolitan occurrence in many permanently frozen alpine and polar settings^11, 35, 59–63^ and they are thought to be important primary producers on ice surfaces,^4, 61^ yet much about their ecosystem function is still unknown. The “true” ice algal species, that are well adapted to temperatures around the freezing point and that would survive in warmer places only temporarily, are the filamentous *Ancylonema nordenskiöldii* Berggren 1871 and the two unicellular species, *Mesotaenium berggrenii* Lagerheim 1892 and *Cylindrocystis brebissonii fo*. Cryophila Kol 1942 (refs 23, 35, 64). These species are all members of the Zygnematales within the Stretophyta, a sister group of the land plants. Whereas *Mesotaenium berggrenii* and *Cylindrocystis brebissonii* have been described in the Arctic^61^ and Antarctica^64^ as well as alpine settings,^60^ *Ancylonema nordenskiöldii* has strikingly so far not been found in the European Alps.

Ice algal taxa are restricted to a very short growth season when liquid water is available and the snow cover is gone. The rest of the year they have to cope with desiccation, darkness and temperature stress. In contrast to snow algae, they have evolved different adaptation strategies, being able to persist in the harsh ice conditions with relatively thin and less rigid cell walls.^59^ For unknown reasons, cysts do not seem to play an important role in ice algae. Cells stay in a vegetative stage and constantly grow and divide during the summer.^42, 65^ *Mesotaenium berggrenii* cells exposed to −25 °C could be revived showing they are also well adapted to overwintering in a frozen state.^64^ Ice algae lack any flagellated and, therefore, motile stage.^66^ They are thus restricted to the ice surface and cannot actively migrate into the snowpack. Recent studies have shown that *Ancylonema nordenskilöldii* and *Mesotaenium berggrenii* accumulate a hydrophilic brownish vascuolar pigment with a tannin nature identified as purpurogallin carboxylic acid-6-O-b-d-glucopyranosidel.^66^ This phenolic purpurogallin derivative has so far only been known from higher plants, further strengthening the close relationship between these algae and land plants.^59^ The brownish pigment has a broad absorption range in the visible as well as the ultraviolet (UV)-A and UV-B range and, therefore, may play an important adaptation role in shielding the chloroplast and avoiding photoinhibition. The same pigmentation has been found in *Mesotaenium berggrenii* cells thriving in less UV irradiated settings, suggesting that this compound may additionally act as an antimicrobial agent in addition to its photoprotective role.^66^ Primary carotenoids have been identified, whereas secondary carotenoids seem to be absent.^60^ Due to their dark brown pigmentation and their wide occurrence, glacial ice algae play an important role in reducing the albedo of bare ice fields during summer and thus in increasing glacial melting.^61, 63^

Compared to what we know about snow algae, the dearth of knowledge on ice algae is striking. One reason could be that they have often been overseen within the similarly coloured cryoconite dust on glacial surfaces.^14^ Another reason could be the lack of representative species in culture collections and our inability so far to grow ice algae in the laboratory.^59^ However, some information can be concluded from other Zygnemataeceae species that can be found in polar and high-alpine soil crusts with high desiccation stress. For instance, in laboratory experiments, these Zygnemataeceae showed increased production of phenolic compounds under UV exposure^67^ and nitrogen limitation induced formation of pre-akinetes,^68^ which showed higher desiccation tolerance than their vegetative stages.^69^ Therefore, both snow and ice algae seem to depend on a range of pigment and fatty acid production to release them from the light, desiccation and nutrient stress experienced on glaciers and ice sheets.

## Cyanobacteria in cryoconite holes

The surfaces of glaciers and ice sheets are pockmarked by characteristic pits known as cryoconite holes (Fig. 2j–i). Cryoconite (reviewed in ref. 13) is a granular mixture of biological and inorganic material that, by reducing albedo and increasing localised melting, forms water filled holes in ice surfaces, a process that has recently been termed biocryomorphology.^21^ Therefore, cryoconite holes are a very different type of habitat on the ice surface as the cryoconite (sediment) material is well submerged in water that is just above the freezing point. The main primary producer in cryoconite holes is cyanobacteria and the filamentous cyanobacterium *Phormidesmis priestleyi*, which is known from Arctic, Alpine and Antarctic environments,^15^ exhibits adaptations that contribute to the development of cryoconite granules.^70^

Perhaps the most fundamental property of filamentous cyanobacteria such as *P*. *priestleyi* that influences interactions with their environment is their ability to release compounds into the environment in the form of extracellular polymeric substances, which in turn include proteins, lipids, polysaccharides and other secondary metabolites. The polysaccharide component (referred to as extracellular polysaccharide) are formed of complex heteropolysaccharides, most of which contain at least six monosaccharides^71^ and can be either released into the environment or bound to cyanobacterial filaments to form a protective sheath or capsule. Both extracellular polymeric substances and extracellular polysaccharides are denominated collectively as ‘‘EPS’’, but for the purposes of this review, EPS refers only to extracellular polysaccharide. The production of EPS in polar cyanobacteria may serve a number of different purposes that assist in survival under the kind of harsh conditions experienced in glacial habitats. For example, EPS is known to act as a cryoprotectant that prevents the disruption of cell membranes by freezing,^72^ can act as a site to deposit UV protective compounds, and may assist in scavenging of metal ions and other nutrients.^71^ In addition to these adaptive properties, EPS can have a substantial influence on the environment and is key to both the formation of cryoconite and stabilisation of microbial biofilms.

**Fig. 3:**
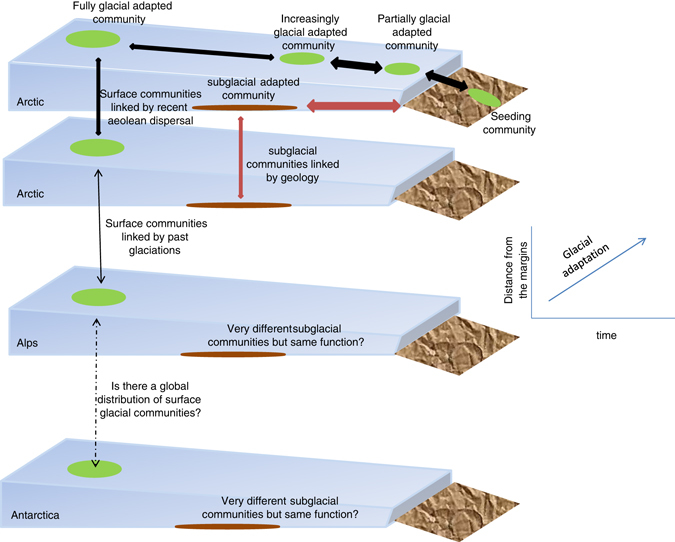
Pathway for microbial adaptation to glacial conditions. Time, size and spatial distance are all likely to contribute to patterns in microbial communities found in glaciers and ice sheets. *Horizontal*/*diagonal arrows* represent selection of microbial communities from glacial margins that are increasingly adapted to glacial survival at the interior of the ice. *Vertical arrows* represent potential links between different glaciers at different spatial and time scales

Rather than being distributed throughout the water column, cryoconite granules are found at the bottom of cryoconite holes, with associated biofilms forming surface attached assemblages rather than forming marine snow like aggregates. During the cryoconite granule formation, cyanobacterial EPS contributes to the aggregation of cryoconite granules by promoting bioflocculation^73^ and acts as a glue that binds together inorganic particles, living microorganisms, and other organic matter in a similar manner to that seen in desert environments.^74^ The overall size of cryoconite granules is determined by the interplay of physical attachment of granules by cyanobacterial filaments, adhesion of particles by EPS, and degradation of organic matter by heterotrophs within and around the granule.^75^ In desert soils where *Microcoleus vaginatus* performs a similar function to *P*. *priestleyi*, the EPS sheath binds strongly to sand particles;^76^ similarly, in cryoconite it is bound EPS rather than released polysaccharides that appears to be most important in determining granule size.^77, 78^ Chemotaxis and motility is also likely to be an important factor in establishing microbe—cryoconite interactions, both in terms of cyanobacterial motility and attachment to cryoconite granules^79^ and chemotaxis of heterotrophs to cyanobacterial exudates.^80^ Overall, such activity is instrumental in collecting material on glacier surfaces into cohesive cryoconite holes that would otherwise be dispersed over wide areas.

As well as influencing the physical structure of cryoconite, cyanobacteria are also of substantial importance to the food web of cryoconite holes, with between 75% and 95% of available carbon in cryoconite having been attributed to cyanobacterial photosynthesis.^81^ Cyanobacterial EPS acts as a substrate that allows for the growth of a complex community of heterotrophs resulting in mature biofilms.^76^ In microbially dominated ecosystems, EPS can represent a major source of biologically labile carbon to fuel the microbial web^22, 82^ and is both consumed by heterotrophs and re-assimilated by cyanobacteria.^83^

Although cyanobacteria are clearly important organisms in cryoconite holes, they appear to represent a relatively small fraction of the microbial community in some diversity studies. In a metagenome of an alpine cryoconite ecosystem, Proteobacteria were dominant numerically with 63.3% of reads being attributed to this phylum. A considerable fraction of assembled contigs were taken up by Bacteriodetes (14%) and Actinobacteria (11.3%) while a much smaller number of contigs were attributed to cyanobacteria (2.5%) (ref. 84). Similar patterns of high proteobacterial abundance and low cyanobacterial abundance were also reported from Arctic and Antarctic cryoconite systems.^85^ This low relative abundance of cyanobacteria in cryoconite holes compared to their impact upon the environment highlights their importance as ecosystem engineers and supports their position as keystone species in these environments.^86^ Most of the cyanobacterial biomass is probably composed of EPS (i.e., a few cyanobacterial cells have a disproportionate impact on the microbial community of cryoconites because of the large amounts of EPS produced).

Like cyanobacteria from other polar environments,^87^ cryoconite cyanobacteria appear to be psychrotolerant rather than psychrophilic, despite the extreme cold. Optimal rates of carbon fixation by cryoconite cyanobacteria were found to be much higher in warmer conditions than the ambient temperature usually experienced by these organisms.^81^ Furthermore, the genome of *P*. *priestleyi* BC1401 revealed no clear indication of the typical genomic hallmarks of cold adaptation^88^ and the mechanism to produce the cyanobacterial sunscreen pigment scytonemin^89^ was absent from the *P*. *priestleyi* BC1401 genome. Yet adaptations to a coldlife style are still likely to exist in these organisms. Changes in fatty acid composition in response to low temperature are known in cyanobacteria^90^ while some cyanobacteria have shown light and temperature acclimation through the production of carotenoids and modification of the photosynthetic apparatus.^91, 92^ However, it is not yet known the extent to which these mechanisms might be operating in cyanobacteria inhabiting cryoconites. Several pathways responsible for EPS biosynthesis were identified in *P*. *priestleyi* BC1401 (ref. 88) and understanding the way these mechanisms are regulated will give us a greater understanding of how cyanobacterial EPS contributes to both cold tolerance and cryoconite formation.

## Bacteria, archaea, fungi and viruses in supraglacial and subglacial environments

### Supraglacial environments

Heterotrophic bacteria are common within the various supraglacial habitats where they play an important role in nutrient cycling from decaying organic matter. Proteobacteria, particularly Alpha- and Beta-Proteobacteria, Bacteroidetes and Actinobacteria dominate the heterotropic communities at the ice surface, yet spatial differences are apparent between different scales. Depending on the spatial scale of comparison, bacterial community composition may differ between snow, ice and cryoconite holes on the same glacier,^11^ between adjacent glaciers^93^ and between polar regions.^85^ Local factors, such as glacier catchment geology,^47^ hydrology,^93^ slope gradient,^21^ bird fertilisation^27^ have all been implicated in explaining variations in microbial community composition between habitats and glaciers. Although known since the 30’s,^94^ a relevant and still debated question is the role that aerially delivered autochthonous biological inputs have on microbial processes in terrestrial icy habitats.^95, 96^ Assessment of magnitudes, rates, diversity or function of aerobiological inputs onto glaciers and ice sheets or the contributions of so delivered microbes to carbon and nutrient cycling in such biomes is poorly quantified. This is primarily because of the lack of standardised or comparable bio-aerosols sampling protocols (passive vs. active, low vs. high air volumes, on filters vs. on agar plates vs. in liquid) for remote Arctic, Antarctica or Alpine locations. Most sampling and analyses approaches are copied from clean room technologies that are not necessarily transferrable to sampling air from remote regions.^6^ A study looking at the bacterial biogeography in cryoconites on various Svalbard glaciers identified a core community (16 out of 755 Operational Taxonomic Units (OTUs) identified in cryoconites) that are present in mean relative abundance >1% per sample.^86^ Those OTUs are also often found in other cryospheric systems. Cyanobacteria *Leptolyngbya* and *Phormidium* were key in Gokul et al.^86^ study demonstrating their importance as cryoconite engineer species (see section above), while among the heterotrophs, the families *Microbacteriaceae* and *Intrasporangiaceae* among the Actinobacteria seem to be key components of cryoconites. Other studies have also identified the genus *Polaromonas* as abundant and important members of glacial ice in Arctic, Antarctic and Alps.^97, 98^ The diversity among *Polaromonas* is large and a comparison of cultures between Arctic and Antarctica shows that, although having similar 16S ribosomal RNA (rRNA) genes, the metabolic traits between the poles are different, suggesting adaptation of the genus to different environmental conditions.^98^ More metagenomic studies of glaciers and ice sheets will provide more opportunities to link diversity and functionality of these habitats. A recent metagenomic study shows that the diversity of functions in cryoconite holes is comparable to any other environment, with a range of metabolic pathways associated with organic carbon degradation and acquisition of nutrients.^84^ A metagenome from Alpine and Himalayan glaciers also revealed high abundance of genes associated with heterotrophic anoxygenic phototrophy.^97^

Archaea have also been detected at the surface of glaciers in snow and ice samples, although the diversity is usually very limited, as the number of studies available or the number of sites of which they are found. Usually when Archaea are found at the surface of glaciers, ammonium-oxidising-Archaea are the dominant group.^7, 29, 99^ Studies of fungi in snow and ice are rare and mostly limited to snow molds, which are not active in the snow itself, but attack dormant plants under the snow cover.^100^ Psychrophilic basidomycetous yeasts have been reported in Arctic,^11, 101^ Antarctic^102^ and alpine settings.^103^ Only recently have *Chytridiomycota* been described as abundant in alpine and Arctic snow,^29, 51, 104^ where they are believed to play important roles in nutrient release through their saprotrophic or parasitic activities^105^ and in snow food-web dynamics.^104^ Nevertheless, their diversity and function in snow are poorly known. Brown et al.^51^ found patterns of co-occurrence between these *Chytridiomycota* and snow algae and suggested that they either share similar environmental tolerance or that the algae act as an environmental filter in fungal community assembly.

There a few studies conducted in supraglacial habitats demonstrating that viruses have an important role in controlling bacterial mortality and potentially the release of labile dissolved organic matter to downstream environments.^106^ Bellas et al.^107^ calculated that there is strong viral shunt in cryoconite holes where viral production seemed to cause nearly all the bacterial mortality of those habitats. This strong top-down control must impose selective pressures on the bacterial community. This has been recently demonstrated by another study in incubations under ambient temperature and nutrient conditions.^108^ On the other hand, Rassner et al.^108^ also demonstrated that addition of nutrients to cryoconite bacterial communities allow for certain members of the community (e.g., the Beta-proteobacteria *Janthinobacterium* sp.) to thrive and escape its viral control. Analyses of putative viral genomes from Svalbard and Greenland cryoconites reveal a range of novel group of viruses, including groups with unusual life strategies and genes to control the replication of their hosts.^109^ There is certainly a lot more to learn on how viruses could control the microbiome of glaciers and ice sheets.

### Subglacial environments

Under temperate and polythermal glaciers, as well as large sections of the Greenland and Antarctic ice sheets, liquid water can also occur due to a combination of basal ice being at the pressure-melting point and energy coming from geothermal and frictional activities. Those subglacial systems have a high rock:water ratio, which generates high amounts of weathering products and nutrients. Weathering can happen abiotically via glacial comminution of the bedrock, generating reactions such as pyrite oxidation and H_2_ production.^110, 111^ Those conditions, in combination with lack of light and redox potential that ranges from well oxygenated to completely anoxic habitats, provide ideal conditions for the development of a consortia of cold-adapted heterotrophic and chemoautotrophic microbes that can further provide additional reactions to accelerate rock weathering under the ice.^112^ For instance, Boyd et al.^112^ detected increased chemolithotrophic activity from *Sideroxydans lithotrophicus*, an iron sulfide-oxidising autotrophic bacterium commonly found in subglacial environments in association with abiotic pyrite oxidation. Close relatives of other bacterial taxa associated with the sulphur and iron cycle are often found in subglacial systems in both subglacial lakes and under glaciers.^113–115^ Examples include genus *Thiobacillus* and *Thiomicrospira*, which have been found in subglacial settings in both Arctic and Antarctica.^116, 117^ Boyd et al.^112^ also demonstrated that increase in sulphate levels in subglacial meltwater, in turn, was associated with increased calcite and dolomite dissolution of those waters.

Sampling of the subglacial microbiome is far more difficult relative to the ice surface due to accessibility to the bedrock 100s of meters or even kilometres of ice (Fig. 1d–e). Examples of ways in which the subglacial environment has so far been sampled include sampling of the subglacial runoff (e.g., ref. 117), or of basal sediments identified within ice caves or at the terminus of glaciers (e.g., ref. 112) and through drilling of long ice cores from the surface to the bed of the ice (e.g., ref. 118). A few studies have used these approaches to provide a snapshot of the subglacial microbial community. Basal ice that is debris-rich usually contains higher nutrient concentrations that in turn, can sustain high bacterial numbers and microbial activity compared to debris-free basal ice.^119^ Microbial diversity under the ice is usually far lower than the diversity found in supraglacial environments. However, the taxa found in subglacial environments quite often match well with their subglacial geochemical environment and are also often close relatives to other organisms found in other cold environments (e.g., ref. 117).

As for the supraglacial environment, few studies have aimed to detect Archaea in subglacial habitats. However, considering the redox conditions found in those habitats and the potential for abundant substrate availability via rock comminution,^111^ methanogenic Archaea should have enough good conditions to thrive. Close relatives to methanogenic Archaea found in permafrost environments have been detected in both Arctic and Antarctica subglacial habitats.^16^ Many studies have not reported for the presence of methanotrophs in subglacial habitats, suggesting that methane may accumulate in subglacial anoxic conditions. However, one study found that the order *Methylococcales*, known to be methanotrophs, were abundant and active in the subglacial runoff of the western margin of the Greenland ice sheet.^120^ This study suggests that nearly all methane produced in the anoxic waters of long-term stored waters in the subglacial habitat is oxidised once they flow through oxygenated drainage channels.

Assemblages of psychrotolerant fungi have also been reported from subglacial settings including the subglacial Lake Vostok accretion ice and the Vostok ice core.^121^ In addition, filamentous *Penicillium* species were found to occur in high abundance in the sediment-rich subglacial ice of three polythermal glaciers in Svalbard.^122^ Fungi enclosed in englacial and subglacial ice will be released during periods of glacial melting and will likely contribute to biogeochemical processes in cold environments. Yet, we do not know to what extend and a gap in our knowledge of fungi in ice settings prevails. There are no studies on viruses from subglacial environments.

## Biogeochemical cycles on glaciers and ice sheets—links with the local and global Earth system

Many recent studies have attempted to measure microbial activities in situ on glacier surfaces and have often found significant levels of primary and secondary carbon production,^14, 61, 123^ nitrogen fixation,^124^ and viral infectivity,^107^ confirming that they have an important role in the biogeochemical cycles of the terrestrial cold biosphere. Comparisons of bacterial production on glacial surfaces between Arctic and Antarctica show that Arctic glaciers seem far more productive than those in Antarctica.^125^ The reasons are probably due to a longer growing season, higher temperatures, and potentially greater input of nutrients via aeolian transport to the Arctic glaciers compared to Antarctica. Simultaneous measurements of primary and secondary production and community respiration on glacial surfaces should be particularly useful to provide evidence of the potential of glaciers surfaces and ice sheets to accumulate organic carbon. However, the lack of measurements during the whole melt season and of integration in measurements between the different types of supraglacial habitats, has led to an inconclusive picture as to whether glaciers and ice sheets are producing new organic matter or mainly consuming and transforming organic matter from external sources. Overall, some very recent laboratory studies indicate that glacial surfaces have the potential to accumulate organic carbon as a result of in situ microbial processes. Musilova et al.^126^ conducted an experiment in which cryoconite debris had all organic carbon removed, by ashing the debris material at 550 °C, and then inoculating it with a small amount of the original microbial community. After exposure of the material to three simulated Greenlandic summers, microbial activity within the cryoconite debris was enough to generate organic carbon accumulation from nearly zero to up to 7 mg of organic carbon per gram of cryoconite material. In another laboratory experiment, Bagshaw et al.^127^ measured the balance between photosynthesis and respiration simulating the closed conditions often experienced in cryoconite holes in Antarctica for almost one year of continuous light exposure (i.e., simulating several summer seasons). Although they found that shorter-term incubations usually resulted in heterotrophy, the longer term incubations showed clear net autotrophy.

Considering the rates of organic matter production and transformation on glaciers are not trivial, it could be hypothesised that the dissolved organic matter signature from glaciers and ice sheets could carry a strong microbial element (e.g., refs 128–130). Due to ice mass loss as a result of global warming, the flux of dissolved organic carbon from glaciers and ice sheets could be the equivalent of half of the annual flux of dissolved organic carbon from the Amazon River by 2050 (ref. 131). This does not take into consideration the potential increase in organic carbon production on the ice surface due to increase in microbial activity and the extent of the ice covered by algae.

In situ measurements of microbial activity in subglacial environments are limited relative to measurements at the surface of the ice and quantification of microbial processes in subglacial habitats is inferred from data of microbial diversity, a range of geochemical signatures in runoff water, incubations in laboratory or at ice margin and modelling. A combination of one or more of those approaches can strengthen the assumption that microbes are important players of biogeochemical transformations in subglacial systems.^11^ Major efforts to constrain the rates of methane production in subglacial systems have been attempted recently because of the obvious importance of methane as a powerful greenhouse gas. Wadham et al.^16^ measured the potential amount of methane produced beneath Antarctica using a combination of laboratory incubations and modelling approaches and found that it could be of the same order of magnitude as the methane stored in Arctic permafrost (i.e., 70–390 Pg C or 1.31–7.28 × 10^14^ m^3^ of methane gas). Similar efforts have not yet been fully conducted for the Greenland ice sheet, but comparison of the rates of methane production and molecular data between the Antarctic and Greenland ice sheets indicate that the potential methane storage in Greenland is much lower which is reflected by the different types of organic carbon available beneath each ice sheet.^16^

## Conclusions and future perspectives

The diversity of the key ecosystem engineers inhabiting glaciers and ice sheets is considered relatively limited. The main primary producers in snow, ice and cryoconite holes seem to be the same across the globe.^15, 47, 132^ Considering that glaciers and ice sheets are simple physical environments formed by very similar mechanisms across the globe, this provides opportunities for studies in microbial population genetics. Lack of molecular data and the restrictions of the morphological classification applied to identify the eukaryotic algae on ice and snow could also impact the conclusion that these organisms seem to have a cosmopolitan distribution.^37^ At the genus level and most often at the species level snow and ice algal taxa occur in the Arctic, Antarctic and the Alps. Whether the cosmopolitan distribution also extends to the sub-species level has not been sufficiently addressed in previous studies and needs to be included in future work. In the northern hemisphere, it is likely that glaciers had some level of continuity between the high arctic and lower latitude mountains during past glaciations, allowing for genetic exchange between cold environments. Likewise, in longer geological timescales (e.g., during the Neoproterozic Snowball Earth ~ 500 million years ago), the whole planet was probably covered by ice.^133^ The use of molecular markers, comparative genomics in a range of different spatial scales (e.g., within glaciers, between adjacent glaciers, between poles) with close relatives from other non-icy environments, metagenomes and molecular clock information could provide new insights into the origins and evolution of the mechanisms for adaptation in snow and ice algae (Fig. 3).

The associated heterotrophic bacterial community is far more diverse and likely influenced by a range of local factors^47, 85^ compared to the primary producers. While there is very little doubt about the primary producers fitness to thrive on glaciers and ice sheets, which evidenced by their ability to produce and accumulate organic matter in these habitats, very few studies have determined how much of the bacterial heterotrophic community is actually active in the supraglacial environment. In a recent study, where co-extracted 16S rDNA and rRNA from cryoconite samples collected at the margins and the interior of the Greenland ice sheet were analysed, Stibal et al.^134^ found very different bulk and active bacterial communities. By comparing the bulk DNA with the potentially active RNA community, Stibal et al.^134^ provided evidence that, although the gene pool in cryoconites is more diverse at the margins, reflecting the contribution of organisms from adjacent ecosystems to the ice, only a fraction of this community seems to be active. On the other hand, the bacterial community in cryoconites collected ca 30 Km from the margin of the ice seems to be represented by almost entirely of active organisms. Similar studies would certainly help to provide insights about the specific microbial community that is intrinsic to the glacial and ice sheet microbiome. In fact, this is one of the main reasons why small valley glaciers need to be differentiated from ice sheets concerning their microbial processes. The former are much more under the influence of transport of a microbial community and nutrients from adjacent ecosystems and a substantial fraction of the community is probably ill equipped to thrive on the ice. Ice sheets operate on longer time scales, which could allow for more opportunities for cold adaptation to occur.
